# Biosynthesis of silver nanoparticles using *Artocarpus elasticus* stem bark extract

**DOI:** 10.1186/s13065-015-0133-0

**Published:** 2015-11-02

**Authors:** Nur Iffah Shafiqah Binti Abdullah, Mansor B. Ahmad, Kamyar Shameli

**Affiliations:** Department of Chemistry, Faculty of Science, Universiti Putra Malaysia, UPM, 43400 Serdang, Selangor Malaysia; Malaysia-Japan International Institute of Technology, Universiti Teknologi Malaysia, Jalan Sultan Yahya Petra (Jalan Semarak), 54100 Kuala Lumpur, Malaysia

**Keywords:** Biosynthesis, *Artocarpus elasticus*, Silver nanoparticles, Stem bark, Transmission electron microscopy

## Abstract

**Background:**

Green approach in synthesizing metal nanoparticles has gain new interest from the researchers as metal nanoparticles were widely applied in medical equipment and household products. The use of plants in the synthesis of nanoparticles emerges as a cost effective and eco-friendly approach. A green synthetic route for the production of stable silver nanoparticles (Ag-NPs) by using aqueous silver nitrate as metal precursor and *Artocarpus elasticus* stem bark extract act both as reductant and stabilizer is being reported for the first time.

**Results:**

The resultant Ag-NPs were characterized by UV–vis spectroscopy, powder X-Ray diffraction, transmission electron microscopy (TEM), scanning electron microscopy (SEM), and Fourier-transform infra-red (FT-IR). The morphological study by TEM and SEM shows resultant Ag-NPs in spherical form with an average size of 5.81 ± 3.80, 6.95 ± 5.50, 12.39 ± 9.51, and 19.74 ± 9.70 nm at 3, 6, 24, and 48 h. Powder X-ray diffraction showed that the particles are crystalline in nature, with a face-centered cubic structure. The FT-IR spectrum shows prominent peaks appeared corresponds to different functional groups involved in synthesizing Ag-NPs.

**Conclusions:**

Ag-NPs were synthesized using a simple and biosynthetic method by using methanolic extract of *A. elasticus* under room temperature, at different reaction time. The diameters of the biosynthesis Ag-NPs depended on the time of reaction. Thus, with the increase of reaction time in the room temperature the size of Ag-NPs increases. From the results obtained in this effort, one can affirm that *A. elasticus* can play an important role in the bioreduction and stabilization of silver ions to Ag-NPs.

**Graphical abstract:**
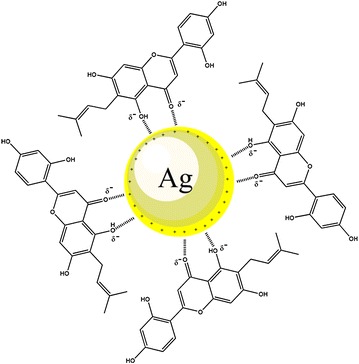
Figure illustrates stabilization of silver nanoparticles after formation by *A. elasticus* stem bark extract.

## Background

Nanotechnology has been emerged as a new technology which design, characterize, produce and applied in the structures, devices and systems by controlling the shape and size at nanometer scale, range from 100 nm down to 1 nm [1].

Metal nanoparticles that have high interest to be synthesized are Ag, Au, Pt and Pb. Silver nanoparticles (Ag-NPs) have the least toxicity to animal cells and highest toxicity to microorganism cells compared to the other metals [2]. Various works have been reported on toxicity of silver nanoparticle against micro-organism such as bacteria [3], fungi [4], viruses [5], and also larvicidal activity [6]. Silver has been widely used in household products such as paint [7], cotton fabrics [8], and in water purification [9]. It was also been applied in surface enhanced raman spectroscopy [10], optical sensor [11], catalyst [12] and in biomedical application [13].

Metal nanoparticles have been synthesized in various techniques in reducing the silver into Ag-NPs including conventional chemical reduction [14], electrochemical [15], irradiation [16, 17], laser ablation [18], polysaccharide [19]. Synthesis of metallic nanoparticles by using living organism is the new approach towards green technology, denominate as biosynthesis.

Biosynthesis of metal nanoparticles includes algae [20], bacteria [21], fungi [22], yeast [23], actinomycetes [24], and plants [25]. From the plant itself, various parts have been explored to give different properties of Ag-NPs. It includes leaf, stem bark, root, flower, vegetable oil, fruit, peel, leaf bud, seed, and callus [26–28]. In addition, biosynthetic process is clearly abiding the three rules of green principles compared to conventional method of chemical reduction.

The *Artocarpus elasticus* (*A. elasticus*) is a distinctive tree in nature, easy to grow, possess anticancer [29, 30], and antimalarial properties [31]. Locals have been using the leaves to nursing mothers, young shoots in curing vomiting blood problems, inner bark used in treating ulcers, and its latex used for dysentery disease [32]. *Artocarpus* are sources of phenolic-derived secondary metabolites which includes flavonoid compounds, particularly of prenylated flavones that exist as the main group of the phenolic constituents [33]. Some of the compounds that have been isolated were artelastin, artelastochromene, artelasticin and artocarpesin [34].

To the best of our knowledge, there is no work reported on Ag-NPs or any other metal nanoparticles synthesized by using *A. elasticus* at ambient temperature. Here, we demonstrate the biosynthesis and characterization of Ag/*A. elasticus* nanoparticles by using silver nitrate and stem bark extract of *A. elasticus*.

## Results and discussion

**Fig. 1:**
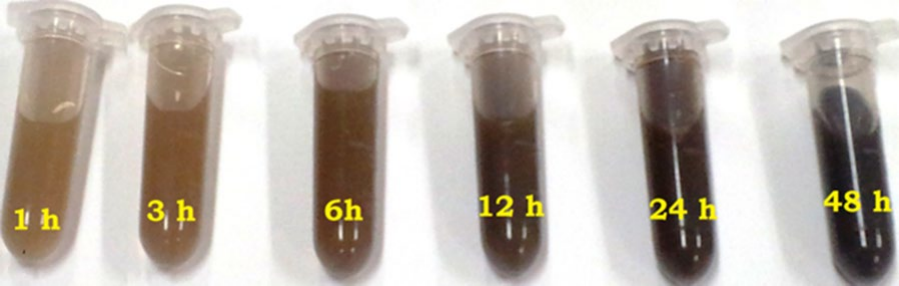
Photograph of synthesized Ag/*A. elasticus* nanoparticles at different reaction time

The reduction of silver ion to Ag-NPs by using *A. elasticus* stem bark extract as both reducing and stabilizing agent and silver nitrate (0.01 M) as a silver precursor was indicated by colour changes of *A. elasticus* extract when incubated with silver nitrate at certain time, as shown in Fig. 1. The solution changed colour from yellow to light brown, and going darker with increasing time (1, 3, 6, 12, 24, and 48 h), at room temperature. It was known that silver nanoparticles colloidal solutions shows intense yellow–brown colour, which occur only in nanoparticles, not in the case of bulk materials due to strong interaction between light and conduction electron of silver in the solution.

The *A. elasticus*with different component and functional groups proved to be able to reduce silver ions to Ag-NPs. The possible chemical equations for synthesizing the Ag-NPs are:

1 $${\text{Ag}}_{{({\text{aq}})}}^{ + } + A. \, elasticus \mathop{\longrightarrow}\limits_{}^{\begin{array}{l}{Stirring}\\{\;at\;Room\;Temp}\end{array}}\;[{\text{Ag}}/A. \, elasticus)]^{+}$$

2 $$[{\text{Ag}}/A. \, elasticus)]^{ + } \mathop{\longrightarrow}\limits_{}^{\begin{array}{l}{Stirring\;for\; 48\,h}\\{\;at\;Room\;Temp}\end{array}}\;[{\text{Ag}}/A. \, elasticus)]$$

After dispersion of silver ions in the *A. elasticus* aqueous solution matrix (Eq. 1), the extract was reacted with the Ag^+^ (aq) to form [Ag/*A. elasticus*)]^+^ complex, which reacted with functional groups of *A. elasticus* components to form [Ag/*A. elasticus*)] (Eq. 2) after left stirred for 48 h [35, 36].

### UV–visible spectroscopy analysis

**Fig. 2:**
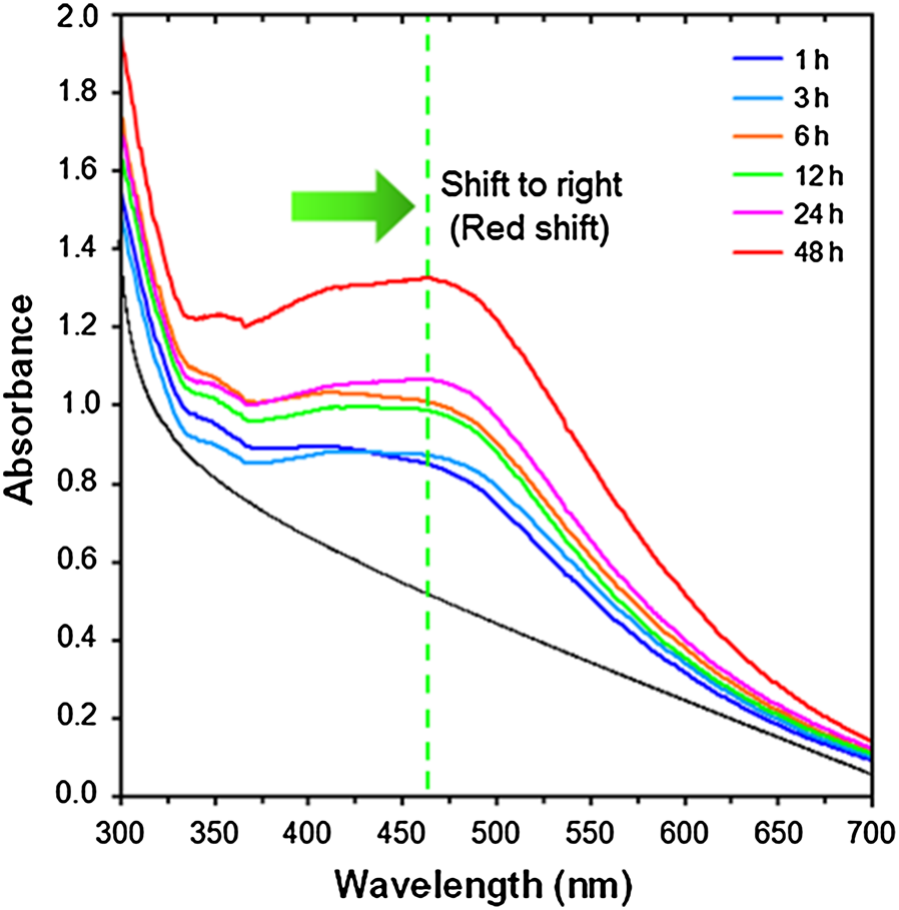
UV-Visible absorption spectra of *A. elasticus* and Ag/*A. elasticus* emulsion prepared at 1, 3, 6, 12, 24, 48 and 72 h

The formation of Ag-NPs was followed by measuring the surface plasmon resonance (SPR) of the *A. elasticus* and Ag/*A. elasticus* emulsions over the wavelength range from 300 to 700 nm. The preparation of Ag-NPs was studied by UV–visible spectroscopy, which has proven to be a useful spectroscopic method for the detection of prepared metallic nanoparticles. It was known that spherical Ag-NPs display a SPR band at around 400–450 nm, depending on its size [37]. The SPR band characteristics of Ag-NPs were detected around 406–460 nm (Fig. 2), which strongly suggests that the Ag-NPs were spherical in shape and have been confirmed by the TEM results of this study. As shown in Fig. 2, the intensity of the SPR peak increased as the reaction time increased, which indicated the continued reduction of the silver ions, and the increase of the absorbance indicates that the concentration of Ag-NPs increases.

At 1 h of reaction time, low intensity of maximum SPR was recorded at 406 nm. However, with increasing time, particles aggregates, causing the conduction electrons near each particle surface become delocalized and shared among neighbouring particles, thus red-shifting the SPR into longer wavelengths from 406 to 424, 420, 433, 455 and 460 nm. At the end of the reaction (48 h), the absorbance was considerably increased and the λ_max_ value was slightly red-shifted to 460 nm, compared with the 24 h reaction time.

At the initial stage of the reaction, the Ag-NPs formed with a narrow size distribution which led to a SPR peak at about 406 nm. After this stage, the Ag-NPs could associate due to increases of reaction time to form bigger size of Ag-NPs. However, at 48 h of reaction time, the absorbance is the largest but also broad compared to the other reaction time, suggesting bigger silver nanoparticles with stable properties. Shoulder peaks were also observed for all of the samples, at 350 nm [38], indicating the existence of bulk silver. Other works presented a broader peak with maximum at 490 nm that indicating larger size of Ag-NPs [39]. However, at 72 h of reaction time, the particles agglomerate, thus showing no distinguishable maximum SPR band. After reaching certain particle size, the plant extract which act as stabilizer was no longer able to withhold the nanoparticles from agglomeration [40].

### Powder X-ray diffraction

**Fig. 3:**
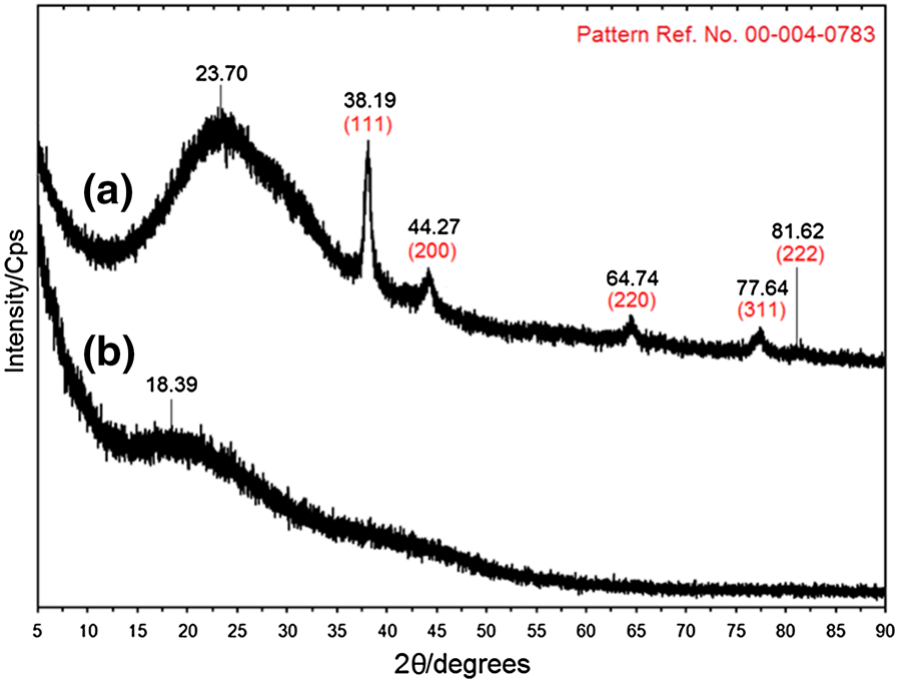
XRD patterns of *a* crude *A. elasticus* *b* synthesized Ag/*A. elasticus* nanoparticles at 48 h

The X-ray diffraction pattern of Ag-NPs synthesized by *A. elasticus* is shown in Fig. 3. The *A. elasticus* pattern shows no peak assign to crystal structure (Fig. 3a). Broad diffraction peak which was centered at 18.39° could be assigned to organic matters in *A. elasticus* extract. After silver nitrate was introduced, the peak shifted to 23.70° (Fig. 3b). The Ag/*A. elasticus* nanoparticles pattern exhibited intense peaks at 38.19°, 44.27°, 64.74°, 77.64° and 81.62° that could be attributed to 111, 200, 220, 311, and 222 crystallographic planes of the face-centered cubic silver crystals, respectively (Powder Diffraction File Card: 00-004-0783) compared to pure silver pattern [41, 42]. There are no other irrelevant peaks observed, indicating only pure crystalline silver exist.

### Morphology study

**Fig. 4:**
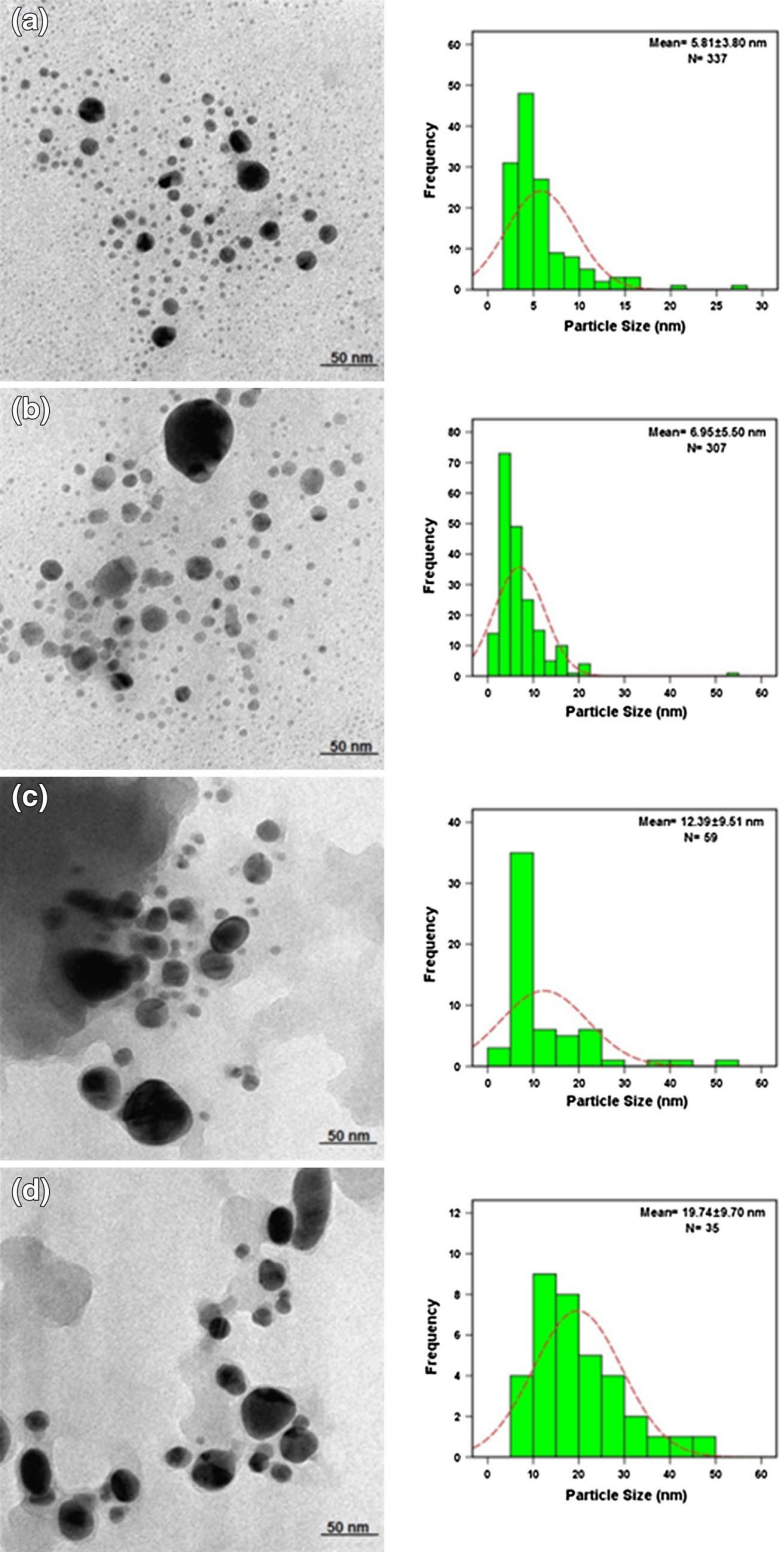
TEM image and histogram of Ag/*A. elasticus* nanoparticles at 3, 6, 24 and 48 h reaction time (**a**–**d**)

TEM images and their size distributions (Fig. 4) show the mean diameters and standard deviation of the Ag/*A. elasticus* nanoparticles as 5.81 ± 3.80, 6.95 ± 5.50, 12.39 ± 9.51, and 19.74 ± 9.70 nm at 3, 6, 24, and 48 h, respectively. It was noted that the size of the nanoparticles increase with increasing time, due to agglomeration of the nanoparticles. At 3 and 6 h of reaction time, the nanoparticles start to develop, indicated by dark clump of nanoparticles together shown on the image taken and proved by SEM image. The reaction completes at 48 h of reaction time.

**Fig. 5:**
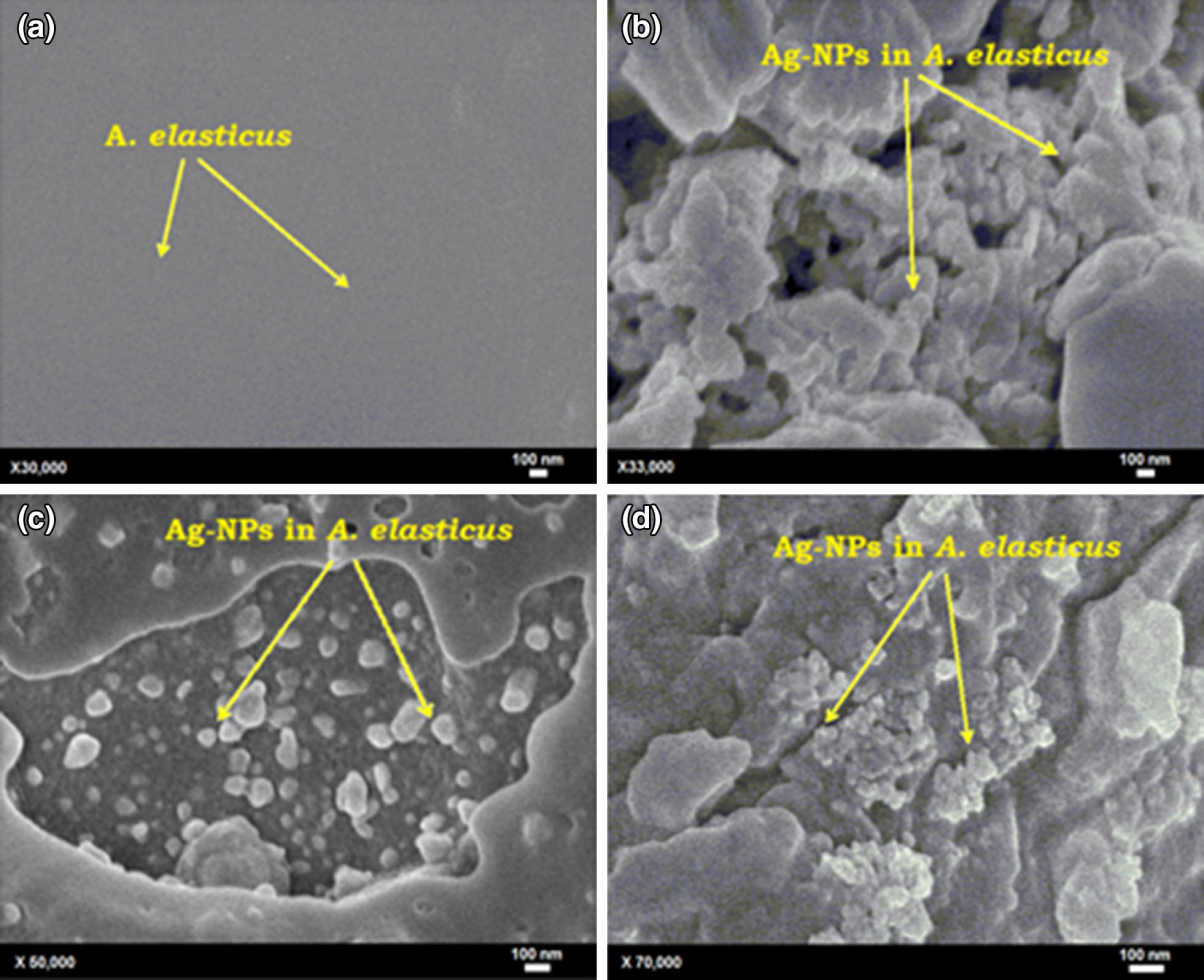
SEM image of **a** crude *A. elasticus,* **b** synthesized Ag/*A. elasticus* nanoparticles at 6, 24 and 48 h reaction time (**a**–**d**)

Figure 5a show scanning electron microscope (SEM) image of a cloudy-like surface of *A. elasticus*. After reacted with AgNO_3_, spherical Ag-NPs had been deposited through reduction by *A. elasticus*. At 6 h reaction time, the nanoparticles start to form as indicated by formation of bulky and near-spherical nanoparticles. Figure 5d distinctly shows that a large quantity of nanoparticles deposited at 48 h reaction time compared to at 6 and 24 h reaction time, as predicted by UV–vis spectrum.

### FT-IR chemical analysis

FT-IR measurements were carried out to identify the possible biomolecules responsible for the reduction; capping and stabilization of the Ag-NPs synthesized using *A. elasticus* extract. For this analysis, solvent was removed to produce Ag/*A. elasticus* nanoparticles powder in order to remove unbound components.

**Fig. 6:**
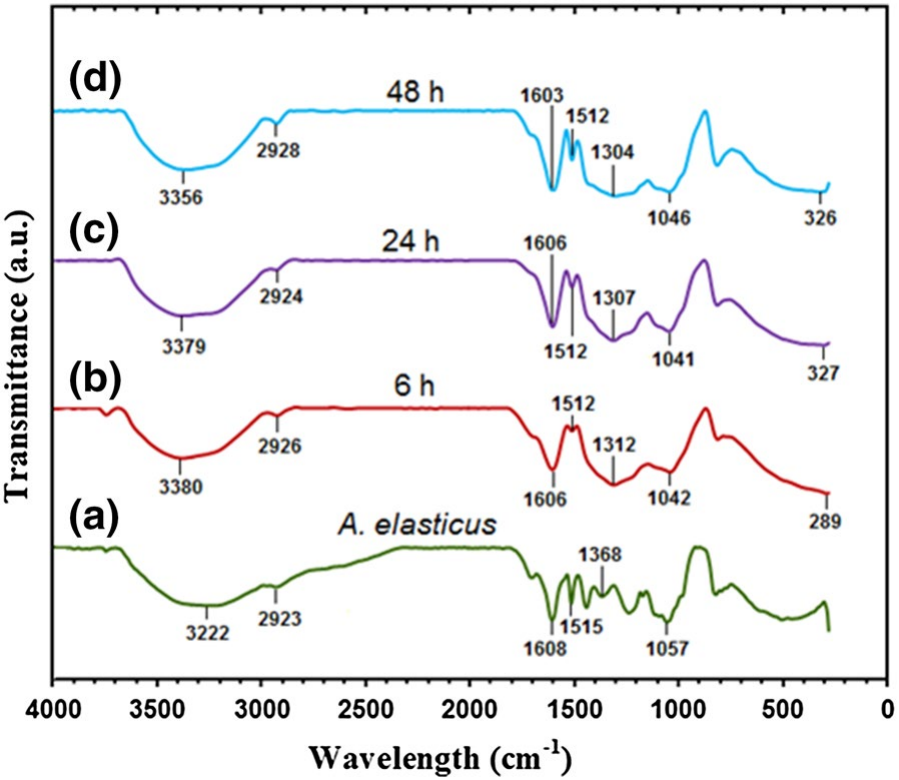
FT-IR spectra of **a** *A. elasticus* crude plant extract, Ag/*A. elasticus* nanoparticles at 6, 24 and 48 h reaction time (**b**–**d**)

The control spectrum (*A. elasticus*) shows numbers of peaks reflecting a complex nature of the compound (Fig. 6a). The peaks corresponding to such bonds such as –C–C–, –C–O–, and –C–O–C– are derived from water soluble phenolic compound of *A. elasticus*. Some shifts in peak position occur to indicate responsibilities of plant extract in reducing and stabilize silver nitrate to Ag/*A. elasticus* nanoparticles. The spectrum of the plant extract shows broad and strong absorbance peak at 3222 cm^−1^ corresponded to O–H stretching. This peak later shift to 3380, 3379 and 3356 cm^−1^ after reacted with silver nitrate at 6, 24 and 48 h, respectively. Peaks at 2926, 2924, and 2928 cm^−1^ are assigned as C-H stretch. In the Fig. 6b–d the broad peaks exist in Ag/*A. elasticus* nanoparticles spectra at 289, 327 and 326 cm^−1^ represents the Ag…O banding with hydroxyl group in *A. elasticus* extract, at 6, 24 and 48 h reaction times respectively [43]. The peaks at 1608, 1515, 1368, 1057 cm^−1^ are shifted to 1603–1606–1606, 1512–1512–1512, 1304–1307–1312, 1046–1041–1042 cm^−1^ respectively in the Ag/*A. elasticus* nanoparticles at 6, 24 and 48 h of reaction time. This shifting indicates the interaction of the nanoparticles with the extract. Flavonoids could be adsorbed on the surface of Ag-NPs, possibly by interaction through hydroxyl group.

## Methods

### Materials

The *A. elasticus* stem barks were collected from Terengganu, Malaysia. Silver nitrate (99.98 %) was purchased from Merck, Germany and used as silver precursor. All reagents used were of analytical grade. All aqueous solutions were prepared using distilled water. All glassware used were cleaned and washed with distilled water and dried before used.

### Extract preparation

The air-dried stem bark was ground into fine powder. The fine powder (400 g) was extracted with 2500 ml of methanol/water overnight at ratio of 70:30 at room temperature. The solution was then filtered; the residue was collected and re-extracted. The solvent then was removed by using rotary vacuum evaporator under vacuum. The concentrated extract was then kept in dark at 4 °C until used.

### Synthesis of Ag/*A. elasticus* nanoparticles

0.5 g of *A. elasticus* was added into 0.01 M aqueous solution of AgNO_3_ (100 ml) with constant stirring at room temperature. Ag-NPs were obtained during the incubation period of 1, 3, 6, 12, 24 and 48 h. Colour changes from light brown to dark brown due to excitation of surface plasmon resonance were observed. The Ag/*A. elasticus* nanoparticles emulsion obtained were kept at 4 °C.

### Characterization methods and instruments

The prepared Ag/*A. elasticus* nanoparticles were characterized by UV–visible spectroscopy, X-ray diffraction (XRD), transmission electron microscopy (TEM), scanning electron microscopy (SEM), and Fourier-transform infrared (FT-IR) spectroscopy. The reduction of silver ions was confirmed by measuring the UV–vis spectrum at 300–700 nm range with UV-1601 Shimadzu, in a glass cuvette. The structures of the Ag-NPs synthesized after 48 h of incubation were examined with using XRD in powder diffractometer, drop coated onto glass substrates. TEM observation of the Ag-NPs prepared was carried out with LEO 912AB EFTEM. The Ag/*A. elasticus* nanoparticle solutions were drop onto copper grid and were analyzed. Morphological characterization of the Ag/*A. elasticus* nanoparticles was performed by Scanning Electron Microscope with using Jeol JSM-7600F Field Emission SEM. The dried powder of Ag/*A. elasticus* nanoparticles were coated on a carbon tape and coated again with gold before subjected to analysis. The FT-IR spectra were recorded in the range of 280-4000 cm^−1^ using FT-IR Perkin-Elmer.
